# Glioma-Derived Exosomes and Their Application as Drug Nanoparticles

**DOI:** 10.3390/ijms252312524

**Published:** 2024-11-21

**Authors:** Serena Mastantuono, Ivana Manini, Carla Di Loreto, Antonio Paolo Beltrami, Marco Vindigni, Daniela Cesselli

**Affiliations:** 1 Department of Medicine, University of Udine, Piazzale S. Maria della Misericordia 15, 33100 Udine, Italy; antonio.beltrami@uniud.it (A.P.B.); daniela.cesselli@uniud.it (D.C.); 2 Department of Pathological Anatomy, University Hospital of Udine, Piazzale S. Maria della Misericordia 15, 33100 Udine, Italy; carla.diloreto@uniud.it; 3 Institute of Clinical Pathology, University Hospital of Udine, Piazzale S. Maria della Misericordia 15, 33100 Udine, Italy; 4 Department of Neurosurgery, University Hospital of Udine, Piazzale S. Maria della Misericordia 15, 33100 Udine, Italy; vindigni.marco@asufc.sanita.fvg.it

**Keywords:** glioblastoma multiforme, tumor microenvironment, exosomes, glioma-derived exosomes

## Abstract

Glioblastoma Multiforme (GBM) is the most aggressive primary tumor of the Central Nervous System (CNS) with a low survival rate. The malignancy of GBM is sustained by a bidirectional crosstalk between tumor cells and the Tumor Microenvironment (TME). This mechanism of intercellular communication is mediated, at least in part, by the release of exosomes. Glioma-Derived Exosomes (GDEs) work, indeed, as potent signaling particles promoting the progression of brain tumors by inducing tumor proliferation, invasion, migration, angiogenesis and resistance to chemotherapy or radiation. Given their nanoscale size, exosomes can cross the blood–brain barrier (BBB), thus becoming not only a promising biomarker to predict diagnosis and prognosis but also a therapeutic target to treat GBM. In this review, we describe the structural and functional characteristics of exosomes and their involvement in GBM development, diagnosis, prognosis and treatment. In addition, we discuss how exosomes can be modified to be used as a therapeutic target/drug delivery system for clinical applications.

## 1. Introduction

Glioblastoma Multiforme (GBM) is the most aggressive primary malignant tumor of the adult Central Nervous System (CNS) [1], with an invariably severe prognosis caused by high proliferative activity, rapid development of drug resistance, broad cellular and molecular heterogeneity and a markedly infiltrative nature of GBM cells [2,3].

The main cell subtype responsible for GBM features is a rare population constituted of self-renewing and highly tumorigenic stem cells called Glioma Stem Cells (GSCs) [4]. GSCs contribute to tumor growth and malignancy through their sustained proliferation, invasion, stimulation of angiogenesis and ability to suppress immune responses and develop drug resistance [5]. Moreover, the Tumor Microenvironment (TME) contributes to tumor growth and aggressiveness. In particular, the GBM microenvironment contains various non-tumoral cell types, as well as Extracellular Matrix (ECM) components and secreted molecules such as growth and differentiation factors [6]. GSCs can remodel the TME, which, in turn, supports tumor growth through a continuous crosstalk that favors disease progression [7].

Exosomes are a subpopulation of Small Extracellular Vesicles (SEVs) of endosomal origin that act as mediators of the crosstalk between tumor cells and TME. SEVs can effectively carry and deliver molecular signals to local and distant recipient cells, promoting the various hallmarks of cancers, including tumor growth, metastasis, invasion, tumor angiogenesis and chemotherapeutic drug resistance [8,9]. Consequently, Tumor cell-derived Exosomes (TEXs) can be considered key factors in the pathways responsible for the biological aggressiveness of tumors.

This review focuses on the role of Glioma-Derived Exosomes (GDEs) in glioma development and progression. After a brief description of the general exosome biogenesis and its contents, we focus our attention specifically on GDEs, finally describing the possibility of modifying/altering GDEs to counteract their ability to support tumor growth by hijacking the interaction between TME and tumor cells.

## 2. Extracellular Vesicles: Exosomes

Extracellular Vesicles (EVs) are a heterogeneous class of lipid-bilayer enclosed particles, naturally released from most human cells under physiological and pathological conditions, and their role in intercellular communication makes them a class of very important components in tumor processes [10]. EVs are broadly classified through different biogenesis pathways and sizes into three groups: apoptotic bodies, microvesicles and exosomes [10,11]. The apoptotic bodies are released by blebbing of the plasma membrane during apoptosis, and their size ranges from 50 to 5000 nm; microvesicles bud from plasma membranes and have a size of 50–1000 nm; finally, exosomes are a class of extracellular vesicles produced by the endocytic process, with a diameter ranging from 30 to 150 nm [10,11,12]. This latter subset of extracellular vesicles, in contrast to the larger ones that are expelled directly from the Plasma Membrane (PM), derives from the intracellular endosomal compartment and is released after the fusion of the endosome with the plasma membrane [13].

**Figure 1:**
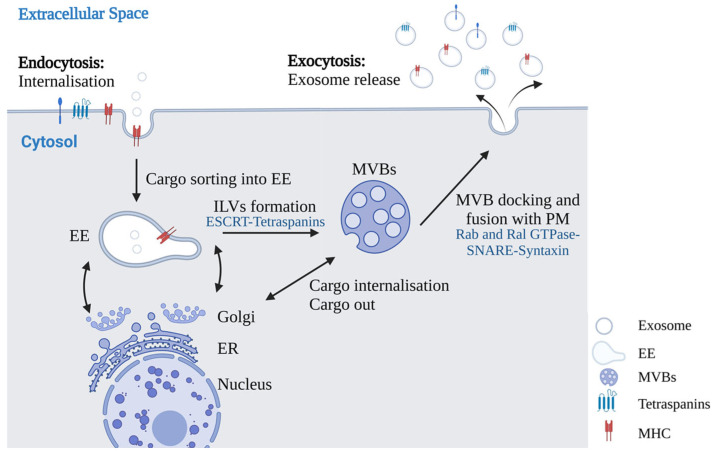
Overview of the exosome’s biogenesis. Cargoes are internalized and sorted into Early Endosome (EE), which then mature into MVBs. Cargoes are also delivered by the trans-Golgi network and possibly by the cytosol. Multivesicular bodies containing cargoes of exosomes are transported to the Plasma Membrane (PM), where they fuse with the cell surface, and ILVs are then secreted as exosomes. Created with BioRender.

Indeed, exosomes originate through a process characterized by an inward invagination of the endosome membrane and the formation of intracellular Multivesicular Bodies (MVBs) containing Intraluminal Vesicles (ILVs) [11]. ILVs are released as exosomes after the fusion of MVB with the plasma membrane [12,13]. The overview of exosome biogenesis is depicted in Figure 1.

To carry out their function, exosomes must bind via receptors to target cells and act either by signaling from the outside or releasing their contents inside the cell by fusion with the membrane, or they can be internalized through endocytosis processes. In any case, exosomes act by modifying the cell’s physiological state and biological function [14,15]. In the case of glioma, it has been shown that EVs secreted by tumor cells participate in tumor growth and spreading [16]. Specifically, it has been shown, in different vitro and in vivo models, that glioma-derived exosomes carry molecules different from those of normal glial cells and that they can facilitate communication between tumor cells and surrounding stromal cells. This communication mechanism, called crosstalk, increases the tumorigenicity of cancer cells and creates a permissive environment in which the tumor can thrive [17].

## 3. GDEs as Mediator in the GBM Tumor Microenvironment

**Figure 2:**
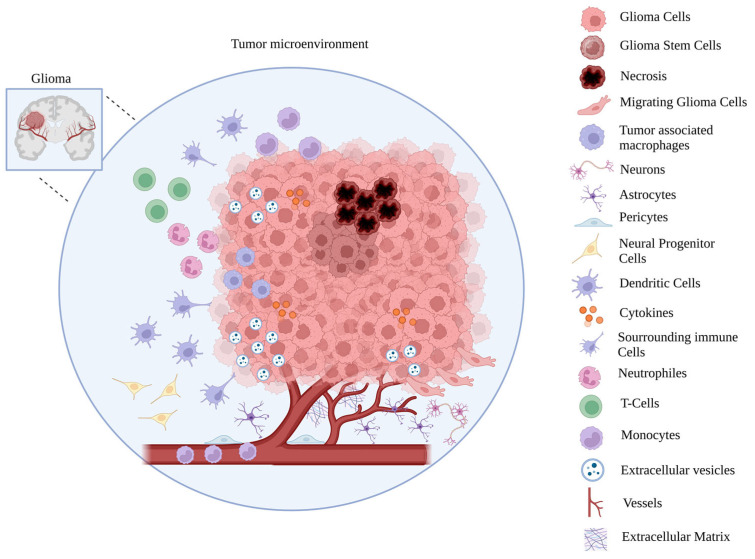
Schematic representation of glioma tumor microenvironment. Created with BioRender.

The GBM tumor microenvironment is composed of different cell populations such as endothelial cells, blood vessel pericytes, microglial cells, T cells, neurons, astrocytes, oligodendrocytes and extracellular matrix components (Figure 2) [18].

Regarding the extracellular matrix, the brain parenchyma is characterized by a particular composition, lacking the fibrillar and rigid matrix composed of collagen, as the intercellular spaces of the brain are filled with proteoglycans, Hyaluronan (HA) and tenascin C, which allow the brain to assume a gelatinous consistency [18,19]. Consequently, the tumor ECM is more condensed and less flexible, making the diffusion of therapeutic agents difficult [20].

It is well accepted that GBM interacts with surrounding non-tumor cells to maintain a microenvironment able to support tumor proliferation, invasion into the brain parenchyma and tumor angiogenesis [7,21]. To better dissect the interaction between tumor cells and their microenvironment, different in vitro models have been developed. In one patient-based in vitro model, it was shown that, from glioma tissue, it is possible to isolate not only GSCs but also a population of non-tumor stem cells named Glioma-Associated Stem Cells (GASCs) [22]. These cells are characterized, in vitro, by a mesenchymal surface immunophenotype and aberrant growth properties, and they are able to stimulate proliferation and self-renewal of glioma stem cells, as well as GSC migration, through the release of exosomes [22,23,24].

Exosomes, GDEs in particular, represent an exceptional communication system because they can deliver not only soluble proteins but also a large group of coding and non-coding RNAs that can profoundly modulate gene expression of the recipient cells [25].

In glioma, the possibility of interfering with this form of intercellular communication requires knowledge of both the mechanism of interaction of GDEs with target cells and the characteristics and function of their biological cargo.

### 3.1. Cellular Recognition of Exosomes

**Figure 3:**
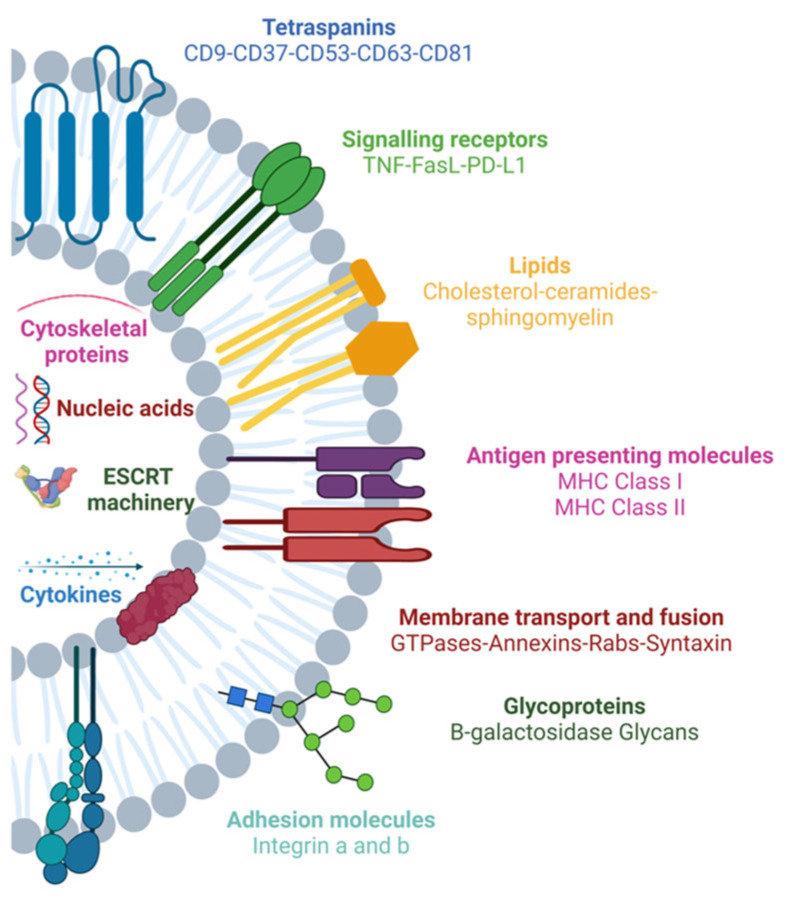
Exosomes are composed of different proteins: transmembrane proteins such as tetraspanins, antigen-presenting molecules, glycoproteins and adhesion molecules; proteins in exosome cargo such as cytoskeletal proteins, Endosomal Sorting Complexes required for Transport (ESCRT) components, membrane transport, fusion proteins and cytokines; nucleic acids; multiple lipids such as cholesterol or ceramides. Created with BioRender.

Exosome surface molecules, such as tetraspanins, immunoglobulins, proteoglycans and lectin receptors, are involved in the binding of exosomes to target cells through mechanisms that are still largely unknown [26,27]. Programmed Death-Ligand 1 (PD-L1), Tumor Necrosis Factor (TNF), Fas Ligand (FasL) and TNF-Related Apoptosis-Inducing Ligand (TRAIL) are the main exosomal ligands and their receptors are located on the surface of tumor cells, becoming potential targets for cancer therapies [27,28] (Figure 3).

#### 3.1.1. Tetraspanins

Tetraspanins are small transmembrane proteins expressed in many species and implicated in different biological processes, such as cell adhesion, motility, proliferation and cancer metastasis [29]. This group of proteins has an important role not only at the level of EV biogenesis but also in EV-mediated cargo transfer [30]. For example, Cluster of Differentiation 63 (CD63) is an important player in exosomal binding and attachment. Yang et al. have demonstrated the possibility of delivering molecules, such as siRNA, across the Blood Brain Barrier (BBB) in vitro and in vivo, using natural exosomes derived from brain endothelial cells as vehicles [31]. Notably, when the exosomes were treated with an antibody blocking CD63, the ability to deliver the cargo to target cells was significantly reduced. In addition, a study by Jennrich et al. further demonstrated the significant role of tetraspanins on the surface of EVs produced in vitro by glioblastoma cells. The expression of the tetraspanin CD9, responsible for the maintenance of glioblastoma-like stem cells [32], and of CD81, associated with enhanced resistance to radiotherapy [33], is increased in low-grade gliomas and glioblastoma, respectively. It was proposed that the upregulation of CD9 expression at the onset of gliomagenesis (corresponding to low-grade glioma) facilitates the accumulation of radio-resistant stem cells. These stem cells up-regulate the DNA damage repair system; next, when a low-grade glioma evolves into a GBM, CD81-mediated DNA repair could prove to be very important in cancer cell survival [34].

#### 3.1.2. Integrins

Integrins are the major group of proteins involved in mediating cell and extracellular matrix attachments. Furthermore, these proteins are involved in cell–cell interactions, which are critical for adhesion, migration and ECM organization [35]. For example, the interactions between integrin Lymphocyte Function-associated Antigen 1 (LFA-1) and Intercellular Adhesion Molecule-1 (ICAM-1) had previously been implicated in the uptake of macrophage exosomes in Brain Microvascular Endothelial Cells (BMECs) comprising the blood–brain barrier [36]. In addition, Integrin β_1_ (ITGB1) is a known EV protein and it is important for invadopodia formation [37]. This protein can interact with other invasion-associated proteins, such as Integrin α_5_ (ITGA5), which dimerizes with ITGB1 to form α_5_β_1,_ which acts as a Fibronectin receptor (FN1) [38,39]. ITGB1 expression is increased in glioblastoma [37], while ITGA5 expression is associated with reduced patient survival [40]. Manini and co-workers demonstrated how Semaphorin7A (SEMA7A), a secreted and membrane-bound glycoprotein, could act as a promigratory stimulus in the glioma microenvironment by interacting with integrins α_1_β_1_ [41]. Specifically, SEMA7A was detected on the surface of exosomes released in vitro by stem cells isolated from the glioma microenvironment. SEMA7A, by interacting with heterodimeric integrins α_1_β_1_ expressed on the GSC surface, could stimulate a rapid Focal Adhesion Kinase (FAK) phosphorylation, increasing the motility of GSC in vitro. Thus, the interaction SEMA7A-β_1_ integrin was identified as a new targetable mediator in the crosstalk occurring in the TME [41].

#### 3.1.3. Proteoglycans

Proteoglycans (PGs) are complex macromolecules characterized by a core protein covalently decorated with linear Glycosaminoglycan (GAG) chains that give rise to PGs of Heparan Sulfate (HSPG) and Chondroitin PG Sulfate (CSPG) [42]. Joshi et al. demonstrated that neural stem cell-derived exosomes can successfully transport their cargo across the BBB using HSPGs as receptors [43]. In particular, Christianson et al. have identified how this receptor plays a key role in the exosomal uptake in U87, a human commercial cell line of glioblastoma multiforme. Internalization is also influenced by the possible presence of fibronectin on the exosomal surface, which can interact with HSPGs, further contributing to the internalization of extracellular vesicles [44]. In addition, Cerezo-Magaña et al. demonstrated that hypoxia-mediated EV uptake depends on increased HSPG endocytosis [42]. Indeed, tumor aggressiveness is associated with a metabolic adaptation by which tumor cells are rescued from hypoxic stress. In this regard, lipid accumulation, as Lipid Droplets (LDs), is a central metabolic phenotype that drives the malignant behavior of several tumor types [45]. Cerezo-Magaña et al. showed that EVs could fuel hypoxic glioma cells to acquire the LD^+^ phenotype, and this effect was associated with an increased EV internalization through HSPG receptors. Consequentially, there was an association between increased internalization of EVs and the LD^+^ malignant phenotype [42].

#### 3.1.4. Lectins

Lectins are a group of proteins that participate in cell–cell communication, facilitating adhesion and intracellular trafficking. These proteins are a population of soluble and membrane-bound receptors that recognize and bind glycan moieties [46]. For example, galectin-3 binding protein is upregulated in different grades of glioma [47]. Rana et al. also identified the overexpression of this protein in plasma exosomes from patients with glioma. For this reason, galectin 3 was suggested as a potential biomarker for the early diagnosis of glioma [48].

### 3.2. Exosomes Content

A variety of molecules have been identified in exosomes, including proteins, lipids and nucleic acids [49]. The double-layer membranes of exosomes protect these molecules from proteases, nucleases and other environmental impacts [50].

Several studies have described the protein, lipid and RNA cargo of exosomes, but less is known about whether and how the cargo is sorted into the vesicles. Indeed, the molecules contained in exosomes are selectively packaged, secreted and transferred to target cells, and they can vary depending on parental cells and pathophysiological conditions [25]. Additionally, exosomes can not only act on adjacent cells but can be transported by body fluids to distant target cells, thus contributing to cancer growth by favoring biological processes such as angiogenesis, infiltration and immune escape [51].

Traditionally, the exosomal protein composition is divided into common and specific proteins [52]. The common proteins exist in almost all exosomes, such as those related to membrane transport and fusion (Rab, Guanosine Triphosphate (GTPases), flotillin), synthesis of multivesicular bodies (ALG-2-Interacting protein X (Alix), Tumor Susceptibility Gene 101 protein (TSG 101)), tetraspanins (CD9, CD63, CD81, CD82) and cytoskeleton proteins (heat shock protein, actin, tubulin) [13,53,54]. The specific proteins are strictly related to the cells of origin. By detecting specific proteins contained in tumor-derived exosomes, it is possible to confirm the origin of exosomes, diagnose related diseases and/or evaluate the effect of treatments [13].

The exosomal lipid composition includes cholesterol, sphingomyelin, phosphatidylserine and ceramide, which constitute a stable membrane structure of exosomes, preserve the exosome morphology and serve as signaling molecules in the intercellular message communication process [55,56].

The exosomal nucleic acids composition is characterized by numerous nucleic acids, such as messenger RNA (mRNA), microRNAs (miRNAs), long non-coding RNAs (lncRNAs) and circular RNAs (circRNAs) [57]. These nucleic acids reflect the status of the source cells, and they can act on recipient cells by modulating gene expression [25].

#### Noncoding RNAs in Exosomes

It has been hypothesized that studying the molecular profile of exosomes produced by a certain tumor can help in defining its type, origin, differentiation and genotype, as well as in predicting its therapeutic response and prognosis [58].

Considering exosomal RNAs, some are tissue-specific, while others are present in exosomes regardless of cellular origin, suggesting potentially different mechanisms related to the sorting of RNA cargoes [58].

Numerous studies have shown that exosomes are enriched with ncRNAs, including microRNA, long-noncoding RNA, circular RNA, piwi-interacting RNAs (piRNAs) and tRNA-derived small noncoding RNA (tsRNA), which play important roles in cancers [59,60].

Thanks to technical advances in the separation and detection of exosomal RNAs, growing evidence shows that exosome-mediated transfer of RNAs between cells is possible and functional. Here, we focus on miRNAs, lncRNAs and circRNAs identified in glioma exosomes.

miRNAs are a group of small non-coding RNAs of about 22–26 nt in length, and their expression in exosomes is proportionally higher than in the source cells [61]. This is explained by the fact that miRNAs enter exosomes earlier than other RNA molecules so that exosome content is enriched in miRNAs [62]. In addition, other studies have shown that the expression levels of exosomal miRNAs secreted by cells depend on the physiological/pathological conditions [62]. Caponnetto et al. found that exosomes isolated from GASCs derived from Low-Grade-Glioma (LGG), characterized by different prognoses, possessed a miRNA content able to modulate key pathways in tumor progression and aggressiveness [63]. Zhu et al. found that the expression level of exosomal miR-21 was significantly increased in the plasma of patients with malignant gliomas compared to healthy individuals [64]. Thuringer et al. showed that miR-5096 derived from exosomes obtained from patients with GBM promoted the growth of filopodium, the invasion of glioma cells by regulating inward rectifier potassium (K^+^) channel Kir4.1, as well as the release of exosomes [65].

lncRNAs are defined as transcripts longer than 200 nt that have no or limited protein-coding capacity. Dysregulation of lncRNA expression has been observed in many human cancers, including gliomas, and they act as tumor suppressors or oncogenes based on the grade and stage of cancers [66]. Exosomal lncRNAs can participate in the onset and progression of gliomas by promoting proliferation, invasion, angiogenesis and drug resistance [67,68].

circRNAs are a novel class of endogenous ncRNAs with a closed loop structure showing tissue- and developmental stage-specific expression [69,70]. Many studies have shown that circRNAs are aberrantly expressed in cancer, including gliomas, and play a vital role in tumor initiation and progression [71,72].

### 3.3. The Role Played by Glioma-Derived Exosomes (GDEs)

GDEs are becoming an important topic in oncology research due to their properties and functional characteristics. Accumulating evidence shows that GDEs are able to transfer biologically active molecules to the cells of the tumor microenvironment [73], thus favoring glioma development and progression as well as drug resistance [74]. The heterogeneous nature of GBM requires the development of more personalized approaches [75]. Consequently, GDEs provide a unique opportunity for the identification and constant monitoring of disease status.

**Figure 4:**
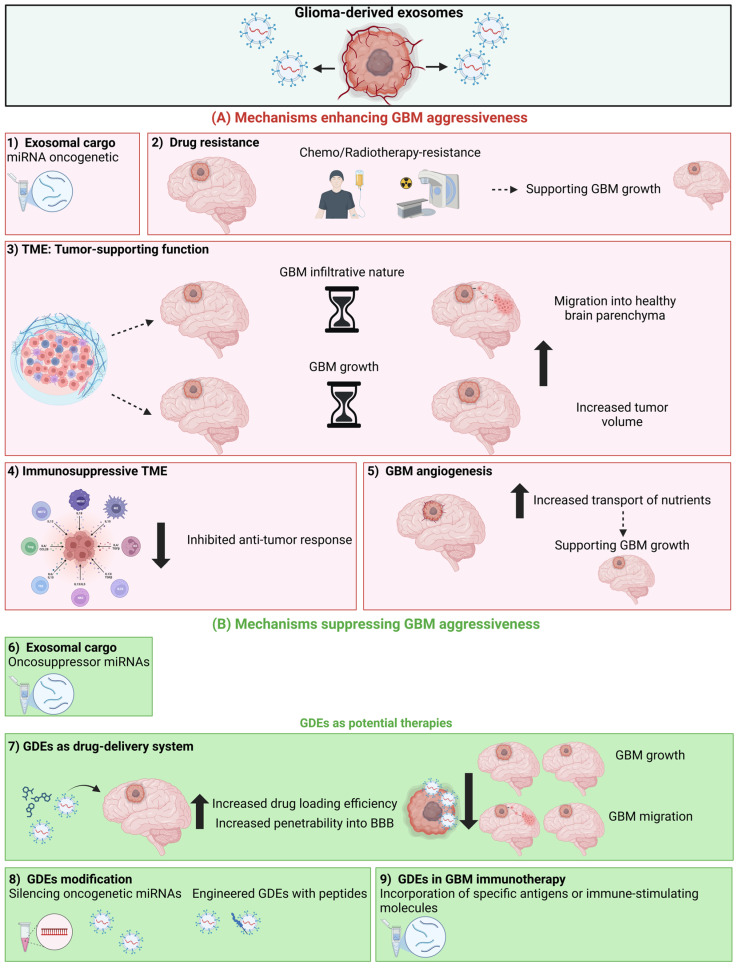
Schematic representation of the mechanisms by which GDEs can enhance GBM aggressiveness (**A**) and how GDEs can be used for tumor suppressive purposes (**B**). Examples of tumor-enhancing mechanisms of GDE are (1) delivery of an exosomal cargo with oncogenetic miRNAs, (2) induction of drug resistance, (3) tumor-supporting function of GDE derived from the TME enhancing glioma cells aggressiveness in terms of tumor growth and infiltrative nature, (4) induction of an immunosuppressive TME and (5) promotion of GBM angiogenesis. Examples of how to exploit exosomes as anti-tumor factors are (6) defining a possible oncosuppressive GDE cargo, (7) using GDE as a drug-delivery system, taking advantage of both their ability to cross the blood–brain barrier and to be highly internalized by glioma cells, (8) silencing oncogenetic GDE cargo or engineering exosomes with peptides, (9) using GDEs in GBM immunotherapy. Created with BioRender.

The role of GDEs in glioma progression is summarized in Figure 4.

#### 3.3.1. The Role of GDEs in Invasion

Two peculiarities of GBM are the mechanisms of invasion and migration, which represent the main reason behind poor prognosis. Indeed, glioma cells tend to invade as single cells that can be found even far from the tumor mass [76]. This makes radical surgery impossible, and the recurrence of the disease and the development of resistance inevitable. Migration and invasion are supported by a continuous crosstalk between tumor cells and tumor microenvironment [77]. Moreover, GDEs exert significant functions in glioma proliferation and invasion through regulating intercellular communication not only in local but also in distant microenvironments [78].

Several studies have investigated the mechanisms through which exosomes exert this pro-infiltrative function, focusing mainly on their cargo. For example, miR-148a contained in exosomes is able to promote cell proliferation and invasion through the activation of the Signal Transducer and Activator of the Transcription 3 (STAT3) pathway, mediated by Cell Adhesion Molecule 1 (CADM1). The inhibition of miR-148a expression in glioma cells significantly inhibited their proliferation [79]. MiR-1246 and miR-10b-5p are upregulated in the Hypoxia Glioma-Derived Exosomes (H-GDEs) and can be transported into normoxic glioma cells, promoting migration and invasion [80]. Thuringer found that glioblastoma-derived exosomal miR-5096 was able to increase the number of motor filamentous pseudopodia, promoting the invasion and migration of GBM cells [68]. Moreover, miR-1587 was found to be highly enriched in glioblastoma-derived exosomes and was able to promote the proliferation of glioma stem-like cells, enhancing their tumorigenicity [81].

In the literature, the role of other ncRNAs has been analyzed in many studies. For example, Han et al. demonstrated that exosomal circ-0001445 upregulates the expression of Sorting Nexin-5 protein (SNX5) that promotes glioma migration and invasion [82]. Similarly, exosomal circZNF652 and exosomal lnc-ATB promote glioma migration and invasion via two different mechanisms. The first, in glioma cells, promotes migration and invasion through the regulation of the miR-486-5p/Serpin family E member 1 (SERPINE1) signaling axis and the epithelial-mesenchymal transition process [83]. The second activates astrocytes and suppresses miR-204-3p expression in an Argonaute-2 (Ago2)-dependent manner, promoting tumor invasive and migratory mechanisms [84].

Alongside this evidence showing how exosomes can transfer oncogenic miRNAs, other works have suggested that selective exosomal packaging and release of miRNAs can occur in cancer cells to bring tumor suppression signaling to the tumor microenvironment. One hypothesis is that, with this mechanism, the tumor cells eliminate factors that would reduce their aggressiveness [85]. In these cases, exosomes are enriched in ncRNAs, which are able to inhibit the growth and invasion of GBM cells. For example, miR-375 expression is downregulated in gliomas, while circulating exosomes of patients with gliomas contain high levels of miR-375. It was shown that miR-375 might be able to inhibit glioma growth by repressing the Cellular Communication Network factor 2 (CCN2 or CTGF)-epidermal growth factor receptor signaling pathway, resulting in the reduction of glioma proliferation, migration and invasion [86]. MiR-7 is one of the most potent tumor suppressors in GBM. Liu et al. showed that miR-7 has an important role in tumor cell proliferation and viability, such as a significant inhibition of GBM xenograft growth in vivo [87]. Additionally, Kefas et al. found that miR-7 was able to inhibit the epidermal growth factor receptor and Protein Kinase B (PKS or Akt) pathways in GBM. Consequently, transfection with miR-7 decreased GBM cell viability and invasiveness [88]. miR-1 is also a tumor suppressor in different types of cancer [89]: Bronisz et al. found that miR-1 could be transferred by GDEs to the surrounding GBM cells, reducing GBM growth and invasion through directly targeting the expression of an important pro-oncogenic factor in GBM, Annexin A2 (ANXA2) [90]. miR-34a is another tumor suppressor. Li et al. showed that miR-34a inhibited brain tumor growth by downregulating c-Met and Notch [91]. Moreover, the overexpression of miR-34a induced apoptosis in GBM cell lines [92]. Sakr et al. showed that transfection of miR-150-5p or miR133a mimics into glioma cell lines reduced Membrane Type 1 Matrix Metalloproteinases (MT1-MMP) expression, thus inhibiting glioma cell proliferation and invasion/migration [93]. Godlewski et al. found that high levels of miR-128 inhibited glioma cell proliferation in vitro and xenograft tumor growth in vivo through the regulation of the *Bm1*-gene [94]. The expression of miR-128 was able to decrease gliomagenesis by downregulating epidermal growth factor receptor and Platelet-Derived Growth Factor Receptor Alpha (PDGFRA) [95].

#### 3.3.2. The Role of GDEs in Angiogenesis

Another important factor in tumorigenesis and aggressiveness is tumor angiogenesis, the development of new blood vessels from existing capillaries or postcapillary venules [96]. An increase in the number of blood vessels leads to an increased transport of nutrients supporting tumor growth [97]. Many studies have revealed that exosomes exert an important function in regulating angiogenesis by delivering ncRNAs in the glioma microenvironment [98]. For example, Wang et al. demonstrated that a specific Vascular Endothelial Growth Factor isoform (VEGF-C) suppresses the Hippo signaling pathway with a strong effect on Tafazzin (TAZ) expression, promoting endothelial cell migration and tubulation [99]. MiR-21 expression is increased in glioblastoma, and it upregulates VEGF expression, promoting the angiogenic capacity of Endothelial Cells (ECs) [100]. In addition, the expression of miR-182-5p and miR148a-3p is also enriched in glioma cell-derived exosomes. Under hypoxic conditions, miR-182-5p directly inhibits Krueppel-Like Factor 2 (KLF2) and KLF4 and induces an accumulation of Vascular Endothelial Growth Factor Receptor (VEGFR) with the consequent promotion of tumor proliferation and angiogenesis. Moreover, it can inhibit tight junction-related proteins and improve vascular permeability [101]. MiR148a-3p activates the epidermal growth factor receptor/Mitogen-Activated Protein Kinase (MAPK) signaling pathway, promoting proliferation and angiogenesis [102]. Moreover, mir-9 could be secreted from glioma cells via exosomes and absorbed by vascular endothelial cells, increasing glioma angiogenesis [98]. Another important role is played by miR-93, able to enhance blood vessel formation by targeting integrin β_8_. Overexpression of miR-93 enhanced angiogenesis in a coculture of human glioblastoma U87 cells with endothelial cells [103]. Li et al. reported that circGLIS3 expression is increased in exosomes derived from High-Grade Glioma (HGG) and it promoted tumor vascularization through the ability to secrete their contents into endothelial cells [104].

#### 3.3.3. The Role of GDEs in Immunosuppressive Microenvironment

GDEs play a critical role in suppressing the antitumor immune response, and they are characterized by a high level of Programmed Death-Ligand 1 (PD-L1) [105]. This molecule binds its ligand Programmed cell Death (PD-1) located on the surface of the activated T-cells and recruits tyrosine phosphatases, Src Homology region 2 domain-containing Phosphatase-1 or -2 (SHP-1/-2), to phosphorylated Immunoreceptor Tyrosine-based Activation Motifs (ITAMs). T Cell Receptor’s (TCR) components are de-phosphorylated and can not transfer activating signals to downstream molecules. Consequently, PD-L1 on the GDEs blocks the activation and proliferation of T cells [106]. Moreover, the uptake of PD-L1 low GDEs increases the levels of Indoleamine 2,3-Dioxygenase (IDO1) and Interleukin 10 (IL-10) mRNAs in recipient cells, encoding immune suppressive proteins [107].

GDEs carry two nucleosidases, CD39 and CD73, on the outer side of the membrane [108]. The uptake of vesicular CD73 by lymphocytes limits their expansion initiated by stimulation with anti-CD3/CD28 mAbs [109]. The main suppressive effect of CD73 is caused by the conversion of 5′ Adenosine Monophosphate (AMP) to adenosine, which binds to the isoform of Adenosine A2A Receptor (A2AR). Signaling through A2AR increases the production of cyclic AMP and dephosphorylation of Signal Transducer and Activator of Transcription 5 (STAT5), negating signals from the IL-2 receptor and TCR in T cells [110].

Galectin-9 (LGALS9), a ligand of CD4 T cell surface molecule T cell Immunoglobulin and Mucin domain containing-3 (TIM-3), is involved in GDE-mediated immune suppression [111]. The binding of Gal-9 to TIM-3 on T cells results in T cell apoptosis. LGALS9/TIM3 signaling pathway regulates T cell functions in several different ways, such as regulation of apoptosis in CD4^+^ T cells and functional exhaustion of CD8^+^ T cells [112].

Azambuja et al. have demonstrated that the internalization of GDEs via macrophages makes them highly susceptible to reprogramming [113]. Moreover, co-incubation with GDEs could trigger the acquisition of a fibroblast shape and the expression of M2 markers, including arginase-1, IL-10, CD206 and Leukocyte Alkaline Phosphatase (LAP) [114].

Studies have reported that GDEs can deliver highly enriched miR-203 to immune cells, promoting immune cell apoptosis, immune escape and rapid tumor growth [115].

Domenis et al. showed that GDEs suppress the T-cell immune response by acting on monocyte maturation rather than by directly interacting with T cells. This mechanism was seen both by analyzing GSC-derived exosomes or exosomes isolated from the plasma of glioma patients. [116]. In this regard, Guo et al. found that tumor exosomal miR-29a and miR-92a stimulated the differentiation of functional Myeloid-Derived Suppressor Cells (MDSCs). MiR-29a and miR-92a silenced HMG Box transcription factor 1 (Hbp1) and cAMP-dependent protein kinase regulatory subunit 1a (Prkar1a) and activated the expansion and activation of myelogenous suppressors cells [117].

In another study, Qian et al. found that miR-1246 in the GDEs bound to the 3′ end of the mRNA of the human *Telomere repeat binding Factor 2 Interacting Protein* (TERF2IP) gene and inhibited its expression through activating the STAT3 signal pathway and inhibiting Nuclear Factor Kappa-light-chain-enhancer of activated B cells (NF-κB) signal pathway [118]. Consequently, the formation of the immunosuppressive tumor microenvironment is promoted.

In a recent work, Tankov et al. demonstrated how hypoxic cancer cells use GDEs to suppress the functions of macrophages, contributing to the immunosuppressive tumor microenvironment. They identified a hypoxia-dependent mechanism able to amplify immunosuppression. Elevated production of miR-25 and miR-93 within hypoxic GBM cells leads to the GDE-shuttled transfer of their cargo to normoxic macrophages, suppressing Cyclic GMP-AMP Synthase (cGAS) expression. GDEs in hypoxic environments contained more miR-25 and miR-93, which were transferred into macrophages. Transfecting with a miR-25 mimetic only inhibited cGAS and type I IFN mRNA production in DNA-challenged macrophages when macrophages were cultured with hypoxic GDEs. Moreover, the addition of hypoxic GDEs induced a significant reduction in the gene expression of Cxc motif chemokine Ligand 9 (Cxcl9), Cxcl10 and IL12b in M1 macrophages. Overall, hypoxic GDEs prevented the efficient polarization of macrophages toward an M1-like state, as measured by a reduced expression of key M1-associated genes [119]. The therapeutic potential of robust cGAS-Stimulator of Interferon Genes (STING) pathway activation in GBM has been demonstrated by the use of synthetic cGAMP as a STING agonist that promotes anti-tumor immunity, implicating both T cells and innate immune cells such as macrophages [120].

#### 3.3.4. The Role of GDEs in Radiation Therapy and in Chemoresistance

Radiotherapy and chemotherapy are key adjuvant strategies for glioma treatment that are able to inhibit the growth of residual tumors and prolong the survival time of patients [1,121]. However, glioma cells rapidly develop resistance, becoming unsusceptible or less sensitive to the action of antitumor therapies [122].

Resistance to radiation therapy is influenced by glioma cell-derived microvesicles, which are involved in glioma development and progression [123]. For example, Dai et al. demonstrated that the antisense transcript of Hypoxia-Inducible Factor 1a (HIF-1a) expression was increased in GBMs promoting invasion and radio-resistance [65]. Yue et al. reported that exosomal miR-301a, secreted by hypoxic GBM cells, can be transferred to normoxic cells and improve cellular radio-resistance [124].

Resistance to chemotherapy is also influenced by glioma cell-derived microvesicles that are involved in tumor development and progression. For example, the expression level of exosome-linked protein Connexin-43 (Cx43) is increased in temozolomide-resistant GBM cells, thus enhancing cell migration and invasion [125]. Set Binding Factor Antisense RNA 1 (SBF2-AS1) expression is upregulated in Temozolomide (TMZ)-resistant GBM cells that secrete exosome-derived SBF2-AS1 and promote X-ray-Repair-Cross-Complementing-4 (XRCC4) expression through binding to miR-151a-3p, thus inducing increased resistance to TMZ [126]. Yang et al. demonstrated that inhibition of miR-221 expression was able to significantly reduce cell proliferation, migration and TMZ resistance [127]. Concerning lncRNAs, Li et al. demonstrated that lnc-TALC can bind to Enolase 1 (ENO1) and promote the phosphorylation of p38 MAPK. Moreover, complement components C5/C5a fractionation is promoted together with TMZ-induced DNA damage repair, inducing chemoresistance [128]. An important role in TMZ resistance is also played by exosome-derived circRNAs. Exosomal circNFIX can enhance resistance to TMZ by binding to miR-132 [129]. Similarly, exosomal circ-HIPK3 can also promote the growth of TMZ-resistant tumor cells by modulating the miR-421/ZIC5 axis [130]. In addition, the expression of exosomal circ-0072083 is increased in TMZ-resistant GBM cells, promoting migration and invasion and inhibiting apoptosis [129].

#### 3.3.5. The Role of GDEs as a Non-Invasive Liquid Biopsy Tool in GBM

Exosomes as liquid biopsy-based tools to assess glioma status and heterogeneity were recently used, together with Circulating Tumor Cells (CTCs) and Cell Free Nucleic Acids (CfNAs), by the Response Assessment in the Neuro Oncology (RANO) working group [131]. GDEs are characterized by a short terminal half-life (around 60 min) [132,133] and can be easily isolated from the patient’s blood, thus allowing a minimally invasive real-time evaluation of the disease’s presence and evolution [134].

Currently, GBM nature is assessed by histological and molecular examination of the tumor tissue. However, surgical sampling fails to describe both the spatial and temporal heterogeneity of the patient tumor [135]. Indeed, surgical sampling is performed at a specific time point, is never radical and can be rarely repeated at tumor relapse [135]. Therefore, the longitudinal monitoring of the disease is usually performed by Magnetic Resonance Imaging (MRI), which is not always reliable to exclude the pseudoprogression of GBM and cannot define changes in the molecular features of the relapsed disease [136,137].

Circulating GDEs could overcome some of these issues. For example, the EV-associated Syndecan-1 (EV SDC1) protein distinguishes high-grade from low-grade glioma patients. In post-operative GBM patients, the EV SDC1 levels in blood plasma changed according to the extent of surgical resection [138]. A short-term strategy could be to combine single exosomal biomarker measurement with tissue biopsy analysis, increasing the accuracy of diagnosis and the reliability of tissue biopsy. On the other hand, in the long term, a panel of EV-associated biomarkers can comprehensively cover the molecular landscape. An additional benefit of the long-term strategy is the reduction in the number and frequency of invasive tissue biopsies, particularly for brain tumors inaccessible to surgical sampling [75].

## 4. Exosomes as Therapeutic Agents

Given the key role played by exosomes in the various mechanisms responsible for the biological aggressiveness of GBM, it is natural to think of exploiting this powerful mechanism of intercellular communication for therapeutic purposes. However, this involves at least two levels of action: 1. choosing which exosomes to use and 2. choosing how to modify them.

Below we will present the possible sources of exosomes, and the mechanisms adopted to modify them.

**Figure 5:**
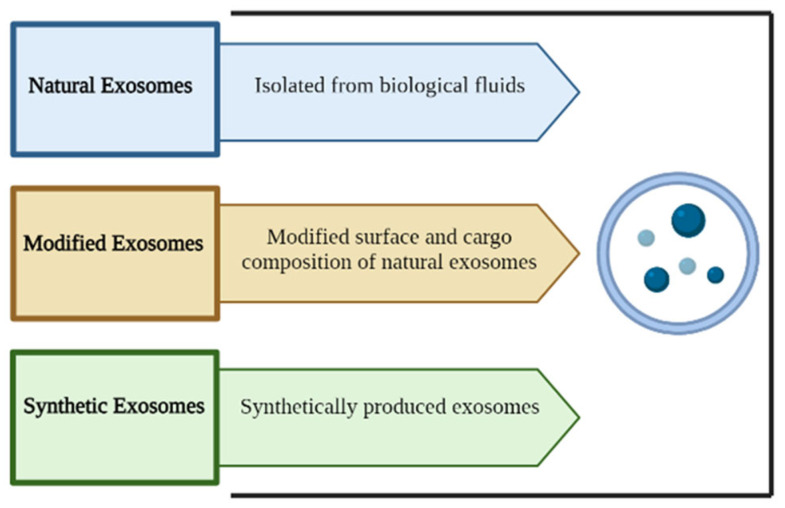
Overview of exosome classification. According to EV origin, exosomes can be divided into natural, modified and synthetic. Created with BioRender.

Exosomes can be classified according to their origin, into natural, modified and synthetic exosomes [139] (Figure 5).

### 4.1. Natural Exosomes

Natural exosomes are secreted from different types of cells, including epithelial cells, endothelial cells, mesenchymal stem cells, macrophages, tumor cells, neurons, dendritic cells, oligodendrocytes, reticulocytes, mast cells and B and T cells [140,141,142,143].

**Figure 6:**
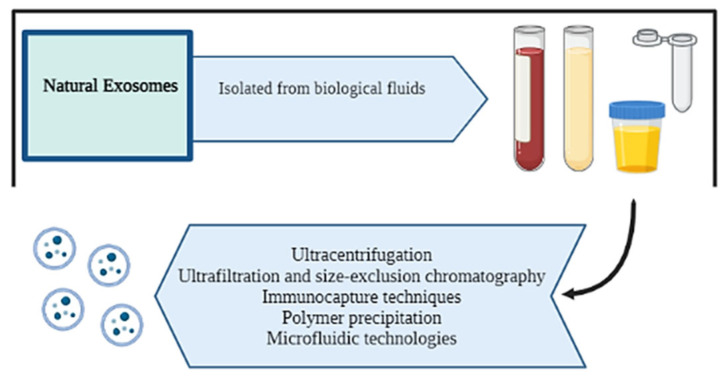
Schematic representation of the different methods for isolating natural exosomes. Created with BioRender.

Various methods have been described to successfully isolate and characterize exosomes from different sources [144] (Figure 6).

Differential ultracentrifugation is the most used isolation technique for isolating animal-derived and plant-derived exosomes [145]. However, other techniques used for isolating natural exosomes are ultrafiltration, size exclusion chromatography, precipitation and microfluidic technologies [146].

### 4.2. Modified Exosomes

Modified exosomes are obtained from natural exosomes subjected to modification for specific therapeutic objectives. Exosomes derived from various natural sources have already been modified in numerous studies, including the incorporation of drugs or the change of the surface charge for faster drug uptake [139].

**Figure 7:**
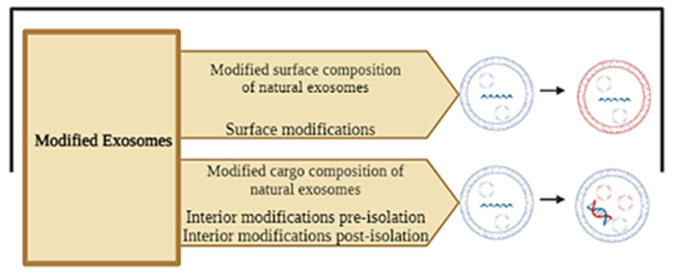
Schematic representation of the two possible exosome modifications: surface or internal modifications. Internal modifications can be distinguished, depending on the timing, in pre-isolation and post-isolation modification methods. Created with BioRender.

The main techniques by which exosomes can be modified are (1) interior modifications, where the composition of the cargo within the exosome is modified, and (2) surface modification, where the structure of the outer surface of the exosomes is modified [147] (Figure 7).

**Figure 8:**
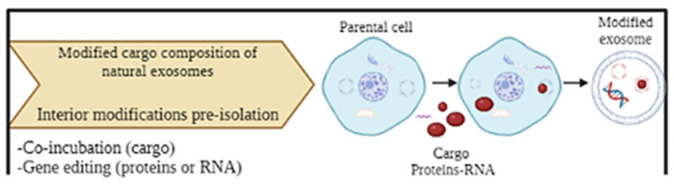
Principle of pre-isolation exosome modification methods: co-incubation and gene editing performed before isolating exosomes from parental cells. Created with BioRender.

Interior modifications represent a group of techniques to incorporate therapeutic agents into the interior of exosomes, ensuring the efficiency and stability of the incorporated cargo [148]. Two types of modifications, depending on the timing in which they are performed, can be identified: pre-isolation and post-isolation modification methods [147]. The main pre-isolation techniques are co-incubation and gene editing, where the modification of exosomes is performed before isolating exosomes from parental cells [140,148,149] (Figure 8).

Co-incubation is a simple method to modify parental cells and encapsulate the desired cargo into the cells. However, it is not possible to control the efficiency of cargo loading [140,148,149]. Gene editing is a method to genetically modify parental cells and to encapsulate the desired cargo, such as RNA or protein [143].

**Figure 9:**
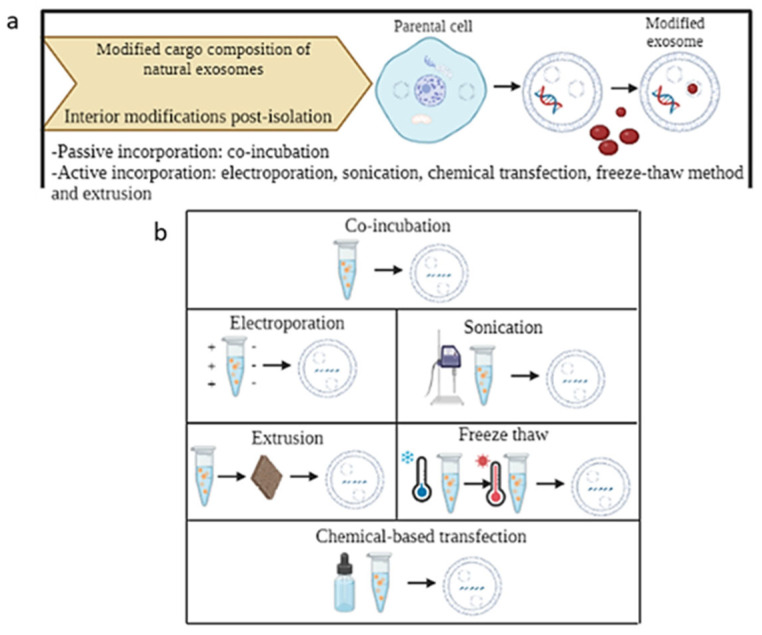
Principle of exosome modification methods post-isolation: passive and active incorporation methods performed after isolating exosomes from parental cells (**a**). Schematic representation of the two possible modifications (**b**). Created with BioRender.

On the other hand, drugs and therapeutic agents can be encapsulated into purified exosomes by post-isolation modification methods directly after their isolation from the cells. This type of modification includes passive or active incorporation methods [150] (Figure 9).

Passive incorporation, a simple and successful method, preserves the morphology of exosomes and ensures diffusion of therapeutic agents into the interior of exosomes through the membrane concentration gradient [150]. Active incorporation is a method able to temporarily disrupt the membrane and encapsulate the cargo into the interior of the exosomes. The membrane integrity of the exosomes is restored when the cargo is encapsulated [145]. Active incorporation includes several techniques: electroporation, sonication, chemical transfection, freeze-thaw method and extrusion [147]. In the electroporation method, pores are provisionally formed in the phospholipid bilayer of exosomes due to the electric field in a conductive solution to allow the entrance of the cargo into the exosomes [140,151]. In the sonication method, the membrane is deformed using ultrasound, and a homogenization probe is used to diffuse the drug into exosomes [152]. In the freeze–thaw method, several cycles of freezing of the exosome preparations at − 80 °C or in liquid nitrogen and re-thawing to room temperature are repeated to ensure the incorporation of drugs [151]. In the chemical transfection, exosomes and cargo are incubated with the surfactant (the most used is saponin) to cause the formation of pores in the membrane and the penetration of drugs [149]. In the extrusion method, a mixture of exosomes and cargo is extruded through a membrane with a pore size between 100 and 400nm using a lipid extruder [140].

**Figure 10:**
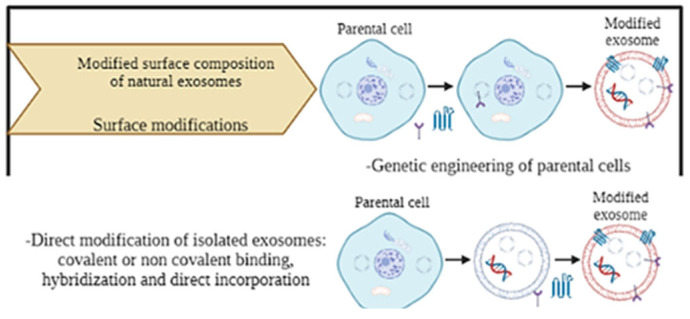
Principle of surface modification methods: gene engineering of parental cells and direct modification of isolated exosomes. Created with BioRender.

Surface modifications are another group of techniques to incorporate therapeutic agents into the interior of exosomes, acting on parental cells or directly on isolated exosomes [140] (Figure 10).

Parental cells can be genetically modified through a viral vector in order to secrete exosomes characterized on their surface by the expression of the peptide resulting from the insertion of the coding sequence within the vector [140,153]. Direct modification of isolated exosomes includes several techniques: covalent binding, non-covalent binding, hybridization and direct incorporation [148]. Covalent binding can be performed through a crosslinking reaction, called click chemistry, where there is a reaction between an alkyl and an azide chemical group to form a stable triazole bond [140,154]. Non-covalent binding includes a multivalent electrostatic approach based on interactions between highly cationic species and negatively charged functional groups on the membrane [154]. In the hybridization method, exosomes are combined with fusogenic liposomes directly, allowing the entry of hydrophobic components [150].

### 4.3. Synthetic Exosomes

**Figure 11:**
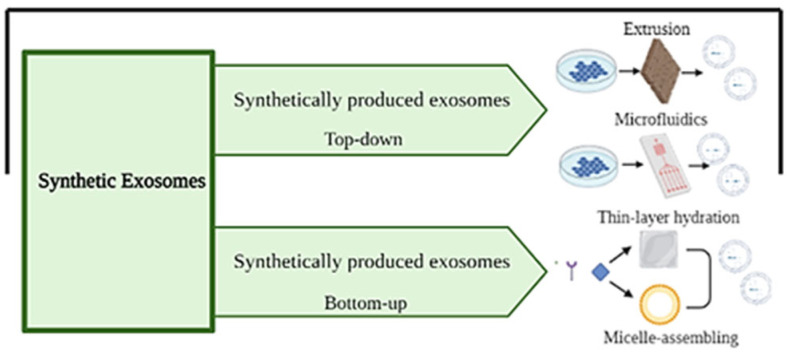
Schematic representation of the two possible modifications: cell-based methodology and lipid membrane bilayer formation methodology incorporation. Created with BioRender.

The properties of exosomes can be mimicked with the production of artificial exosomes, although our knowledge of this possibility is still limited. Synthetic exosomes can be obtained by two methods: cell-based methodology and lipid membrane bilayer formation methodology incorporation [147] (Figure 11).

In the cell-based methodology, cells are broken into small membrane fragments which are assembled into spherical membrane vesicles with the same characteristics as the initial cell [139,155]. Synthetic exosomes can be obtained by extruding cells through a series of polycarbonate membrane filters with reduced pore size or by pressurizing live cells in microfluidic systems [139,152]. On the other hand, in the lipid membrane bilayer formation methodology, the desired cargo can also be incorporated with the thin-film hydration method or reverse-phase evaporation method [139,152,156].

As we will see, to date, most of the experimental evidence obtained has focused on the use of modified natural exosomes. This implies the key role played by natural exosome isolation techniques.

## 5. GDEs Modification: State of the Art

In the context of therapeutic strategies, exosomes can be considered very interesting carriers able to provide efficient drug delivery thanks to their morphology, size and expression of surface markers that allow interaction with target cells.

In particular, the use of glioblastoma-derived exosomes as drug vehicles is further reinforced by their ability to cross the blood–brain barrier, acting as mediators between the tumor and its microenvironment. However, as has been seen, exosomes represent a complex system, and the use of GDE is hampered by several technical problems, such as the low isolation yield and the drug payload strategies, which can reduce their pharmaceutical feasibility [145]. Nonetheless, the possibility of taking advantage of exosomes as targeted vehicles for drug delivery has been recently strengthened by the progress of biotechnology [9,145,157].

Here, we described ten examples of modifications that could improve the therapeutic efficacy of GDEs (Table 1): the use of exosomes as a drug delivery system to incorporate anticancer drugs, such as Paclitaxel (PTX) (1), PTX and Doxorubicin (DOX) (2), TMZ and EPZ015666 (3), selumetinib (4), the engineering of exosomes with peptides to improve the efficiency of loading and transport of chemotherapeutic agents (5), the functionalization of exosomes with transferrin through a click chemistry reaction (6), the application of exosomes in GBM immunotherapy (7), the use of exosomes in vaccination as an autologous source of tumor-associated antigens (8), the application of an engineered platform to potentiate chemotherapy and photoimmunotherapy (9) and the engineering of exosomes conjugated to magnetic particles to disintegrate the defensive axis of ferroptosis (10).

Four of the examples reported describe the use of GDEs as a drug transport system aimed at increasing the drug’s cytotoxic effect. Two examples include surface modifications to increase exosome internalization and BBB crossing. Two examples include GDE modifications for application in immunotherapy. Finally, two examples describe the engineering of GDEs by functionalizing their surface.

### 5.1. GDEs as Drug Delivery System in GBM Therapy: Different Techniques for Loading Anti-Cancer Agents

Since the use of exosomes as drug delivery devices can help to overcome the limitations of other systems, such as biocompatibility and cell penetration, it is mandatory to identify the best methods to efficiently and specifically load and vehiculate molecules and drugs against tumor cells.

Paclitaxel is a powerful mitotic inhibitor anticancer drug used in the treatment of many human cancers, including ovarian, breast and lung cancer [168]. However, due to its chemical nature, PTX is unable to pass through the blood–brain barrier [169,170], and several attempts have been performed and are still ongoing to solve the problem of low bioavailability by loading the drug into positive-charged solid lipid nanoparticles [171] or by disrupting the BBB [172].

In the study by Salarpour et al., two methods to incorporate paclitaxel into U87-derived exosomes were compared [158]. The authors used two techniques for GDE loading: incubation for 1 h at 37 °C and sonication with an ultrasonic probe. The authors showed the superiority of sonication in terms of the amount of incorporated PTX and subsequent cytotoxicity on U87. However, the sonication method caused an enlargement of the exosome’s diameter, which may be ascribed to the reorganization of the membrane vesicles [158].

Another problem is the low loading efficiency of anticancer drugs, such as PTX or doxorubicin, into exosomes. To improve the loading efficiency of drugs, a microfluidic device (Exo-Load) was developed by Thakur and co-workers. In particular, the use of saponin with DOX and PTX to improve its loading efficiency into GDEs combined with increased permeabilization and shear stress-induced stimulation in the microchannel of the Exo-Load microfluidic device optimized drug loading. Next, to increase the exosomal delivery of DOX and PTX to glioma, autologous exosomes were modified via Exo-Load. Consequently, the use of the microfluidic device for loading anticancer agents into exosomes reduced tumor proliferation [159].

Araujo-Abad and co-workers improved the drug delivery system in GBM by testing two procedures to load GDEs with two chemotherapy drugs, such asTMZ and EPZ015666 [160]. TMZ is a DNA alkylating agent that is able to act over the methylation of guanine and adenine bases and break double-stranded DNA, causing cell cycle arrest [173]. EPZ015666 is a small molecule able to compete with the substrate-binding pocket of the arginine methyltransferase-5 (PRMT5), thus inhibiting it [174]. The first method used to load GDEs, called indirect incubation [175], consists of treating the cells for at least 48 h with high doses of drug before collecting the released GDEs [160]. In the second method, named direct incubation [176], isolated GDEs were incubated in a medium containing a low dosage of the drug [160]. As a result, in both systems, GDEs were successfully loaded with drugs. However, direct incubation was more efficient due to better interaction with the lipid bilayer of GDE membranes. The drug-loaded GDEs were able to exert their cytotoxic effect and reduce cell proliferation in vitro [160]. This work offers a great therapeutic opportunity with the possibility to use GDEs as drug delivery systems for GBM treatment, even at low doses of chemotherapeutic drugs.

Lee et al. have demonstrated the effects of exosomes derived from tumor cells and loaded with selumetinib [161]. Selumetinib is a new anticancer drug used for the treatment of Neurofibromatosis type 1 (NF type 1)-related plexiform neurofibromas. In GBM, NF type 1 is highly mutated, and, consequently, this drug can be considered a good candidate for the treatment of GBM [177]. Selumetinib-loaded U87-derived exosomes (U87-Selu EXOs) were obtained by the electroporation method. In vivo, a male Balb/c-nude mouse treated with A549-Selu EXOs showed an increase in volume up to day 8, while, in the group treated with U87-Selu EXOs, the tumor volume increased slightly until it decreased after 4 days. In particular, U87-Selu EXOs induced cell apoptosis, increasing the level of Poly (ADP-ribose) Polymerase (PARP) and decreasing the level of B-Cell Lymphoma 2 (Bcl2). Through a Fluorescence-Activated Cell Sorting (FACS) analysis, the apoptosis hypothesis was confirmed by cell arrest in the G1 phases. G1 phase arrest is associated with apoptosis of mesenchymal or epithelial cells [178,179]. In both the U87-Selu EXOs and U87 treated with selumetinib, there was a reduction in the expression level of Phosphor Mitogen-activated protein Kinase (pMEK) due to the fact that this drug is a specific inhibitor of pMEK [161]. As a result, the antitumor mechanism of U87-Selu EXOs is similar to that of selumetinib even after drug loading into vesicles [180,181].

### 5.2. Surface Engineered GDEs to Cross BBB

The use of GDEs in therapies may be hampered by low penetrability into the blood–brain barrier. Consequently, surface-functionalized GDEs are intended to increase their ability to cross the BBB, thus allowing their antitumor function.

In a study performed by Zhou et al., a simple strategy to engineer GBM cell-derived exosomes, named Exo@TDPs, was described [162]. Through this approach, it was possible to modify GDEs to improve both biosafety and delivery of the chemotherapeutic agents TMZ and DOX into GBM tissues. First, they reduced the potential pro-tumoral risk of tumor-derived exosomes by favoring the loss of the endogenous cargo using a sonication method, thus improving the level of biosafety. Then, engineered GDEs were decorated with both Angiopep-2 (Ang-2) and Cluster of Differentiation 133 (CD133)-targeted peptides and loaded with TMZ and DOX. Peptides constitute an important class of possible candidates as targeting molecules. Indeed, Ang-2 and CD133 were included to improve the ability to penetrate the BBB and target tumor cells [162]. Ang-2 possesses a high affinity for low-density Lipoprotein Receptor-related Protein 1 (LRP-1), which is highly expressed in both BBB endothelial cells and GBM tissues [182]. The CD133-positive human brain tumor cells constitute a population of GBM-initiating stem cells [183]. Compared with the free TMZ or DOX, Exo@TDPs showed a sustained drug release profile in which both TMZ and DOX were gradually released, demonstrating that chemotherapeutic agents were sheltered by the Exo@TDPs, thus avoiding undesirable drug release into the blood circulation. The targeting capability of Exo@TDPs on patient-derived glioma stem cells was confirmed using tumor sphere formation assays. Moreover, in an in vitro BBB model, Exo@TDPs, thanks to the presence of Ang-2 and CD133 targeted peptides, showed an excellent ability to cross the BBB and to deliver TMZ and DOX with a preferential affinity for glioma cells. Consequently, Exo@TDPs were suggested as a promising tool to enhance the BBB permeability to chemotherapeutic agents and to target cancer cells, thus exerting an antitumor effect. Indeed, when Exo@TDPs were intravenously injected into the BALB/c mice bearing wild-type U251 GBM tumors in the brain, they were found in the deep tumor parenchyma 24 h after injection, and their homing efficiency was higher with respect to EXO not decorated by Ang2/CD133-targeted peptides. Accordingly, with respect to TMZ and DOX alone or EXO not modified by targeted peptides, EXO@TDPs demonstrated a significant increase in both inhibition of tumor growth and mice overall survival [162].

Yang and co-workers developed a DOX&siTGF-β@ACTE (Ds@ACTE) platform designed to specifically recognize the Transferrin (Tf) Receptor (TfR) on the blood–brain barrier [163]. An Acid-Cleavable Transferrin decorated engineering Exosome (ACTE)-based brain targeting delivery system was able to deliver small interference RNA towards Transform Growth Factor-β (siTGF-β) and DOX to GBM site for combination chemo-immunotherapy. Consequently, DOX&siTGF-β@Exo (Ds@Exo) was separated from the Tf-TfR complex, enhancing BBB transcytosis. After crossing the BBB, the separated Ds@Exo can target GBM cells via the homing effect. Moreover, in vivo studies showed significant downregulation of Transforming Growth Factor-β (TGF-β) expression, reprogramming the immunosuppressive microenvironment [163].

### 5.3. GDEs Surface Modification for Immunotherapy

GDEs can interact with immune cells and modulate immune responses in the TME. Incorporating specific antigens or immune-stimulating molecules into GDEs, immune cells could be activated, thus inducing an anti-tumor immune response.

For example, in Liu’s study, the specific anti-tumor immune response was induced by co-delivery of tumor-derived exosomes with a-Galactosylceramide (α-GalCer) on Dendritic Cell (DC)-based immunotherapy for GBM [164). Invariant Natural Killer T (iNKT) cells are a special subpopulation of T cells able to express uniform activated T cell receptors and to proliferate via recognizing a specific glycolipid antigen α-GalCer [184]. GDEs were utilized as a more potent antigen to load DCs. The co-delivery of GDEs with α-GalCer-pulsed DCs modified in the tumor microenvironment the balance between the release of immunoinhibitory and immunostimulatory factors, favoring the second ones. Moreover, GDEs combined with iNKT cells broke immune tolerance, inducing a strong antigen-specific Cytotoxic T Lymphocyte (CTL) response against GBM cells. Finally, they performed an in vivo immunotherapy experiment using orthotopic glioblastoma-bearing rats and they showed how there was an effective immune response with prolonged survival when rats were treated with α-GalCer-pulsed DCs [164].

Recently, total tumor lysate-pulsed dendritic cell vaccination has been currently evaluated as a therapy for GBM [185]. Since immunization of glioblastoma patients with highly personalized DC vaccination would appear to be a promising approach to boost CD4^+^ and CD8^+^ T-cell responses, EVs may constitute a more comprehensive, cell-free, autologous source of neo-antigens and tumor-associated antigens to pulse dendritic cells and induce the initiation of an antitumor immune response [186]. In this regard, the capture and uptake of GDEs by DCs would seem to be related to a glycan-dependent mechanism [185,186]. Dusowa et al. have characterized the glycocalyx composition of glioblastoma EVs by lectin-binding ELISA and comprehensive immunogold transmission electron microscopy. As a result, the surface glycan profile of glioblastoma-derived EVs was characterized by α-2,3- and α-2,6 linked sialic acid-capped complex N-glycans and bi-antennary N-glycans [165]. Sialic acids can trigger immune inhibitory sialic acid-binding Ig-like lectin (Siglec) receptors. Consequently, the authors verified on glioblastoma EV the expression of Siglec ligands, which are able to bind dendritic cells (DCs) and trigger an inhibitory stimulus. Conversely, GDEs lack glycans that could bind dendritic cell-specific intercellular adhesion molecule-3-grabbing non-integrin (DC-SIGN, CD209), a receptor that mediates uptake and induction of CD4^+^ and CD8^+^ T cell activation [187]. The authors hypothesized a strategy to modify the surface of the EV glycan to induce a reduction in immune inhibitory Siglec binding. Specifically, they showed that, by desialylation with a pan-sialic acid hydrolase, was possible to reduce the immune inhibitory Siglec binding, while, by insertion of a high-affinity ligand (LewisY) for DC-SIGN, GDE internalization was enhanced by DCs in a DC-SIGN dependent manner [187].

### 5.4. Therapeutic Engineered GDEs

An approach to engineering GDEs in immunotherapy is described in Li’s study [166]. They designed a ginsenoside Rg3 (Rg3)-modified GL261 cell-derived exosome co-loaded with arsenic trioxide and chlorin e6, improving GBM immunotherapy. Rg3 is able to induce significant immunomodulatory effects on remodeling the TME by inducing the polarization of macrophages from M2 to M1 [188]. Arsenic trioxide induces chemotherapy, and chlorin e6 generates ROS under laser irradiation, triggering photodynamic therapy-induced immunogenic cell death. As a result, this construct inhibited orthotopic GBM growth, induced macrophage polarization and facilitated cytotoxic T lymphocyte infiltration [166].

A final example of engineered GDEs is related to magnetic nanoparticles to exploit ferroptosis as a GBM therapy. Ferroptosis is an iron-dependent form of non-apoptotic cell death that can occur through two major pathways: the extrinsic or transporter-dependent pathway and the intrinsic or enzyme-regulated pathway [189]. This newly discovered type of programmed cell death is opening new perspectives for the treatment of GBM. Recently, new drugs able to induce ferroptosis were developed, even though their application is hindered by poor blood–brain barrier penetration and reduced tumor-targeting ability. It has been shown that, to finally induce ferroptosis, both Glutathione Peroxidase 4 (GPX4) and Dihydroorotate Dehydrogenase (DHOHD) must be disrupted. Li and co-workers have proposed, as a therapy for GBM, a novel composite therapeutic platform coupling Magnetic Nanoparticles (MNPs) and engineered exosomes. MNPs were used to take advantage of their magnetic targeting and delivery properties, while exosomes were used to cross the BBB and to deliver specific siRNAs. The MNP was constituted by a Fe_3_O_4_ core and a mesoporous silica shell whose surface was incubated with brequinar (BQR), an inhibitor of DHODH conjugated with a CD63 antibody able to recognize the exosome component. This latter consisted of EVs produced by Human Mesenchymal Stem Cells (hMSCs) modified to express on their surface an ANG peptide (ANG-EXO) and loaded, by electroporation, with small interfering RNA (siRNA) of GPX4 (siGPX4). This system holds characteristics and drug delivery properties of magnetic nanoparticles with the BBB penetration capabilities and siRNA encapsulation properties of engineered exosomes [167]. This MNP@BQR@ANG-EXO-siGPX4 platform, when injected into the tail vein of a human-derived tumor xenograft GBM model, could be preferentially enriched in the brain vasculature by using a helmet with permanent magnets. Indeed, after the application of a local magnetic field, the MNP@BQR@ANG-EXO-siGPX4 platform was enriched in the blood vessels of the brain, followed by the penetration of the BBB by the recognition of the ANG-targeted peptide by low-density LRP-1 receptors. Finally, the GBM synergistic treatment through the combined triple actions of disintegration DHODH and the GPX4 ferroptosis defense mechanism and Fe_3_O_4_ NPs-mediated Fe^2+^ release was able to increase mouse survival [167,189].

## 6. Conclusions

Exosomes are rapidly becoming a new field of interest for researchers worldwide due to their involvement in intercellular communication. In glioma, GDEs are able to efficiently interact with both tumor cells and microenvironmental cells, thus sustaining a crosstalk that supports tumor growth and invasion. In this regard, we highlighted, in this review, the biological properties of exosomes, and we exploited the possibility of modifying them for therapeutic purposes. In particular, we focused our attention on EV biogenesis, release and uptake mechanism, as well as on the strategies that have been developed to interact either with the surface structure or the molecular cargo of exosomes to obtain engineered exosomes with high cell targeting, loading efficiency and desired biological activity.

In GBM, the level of complexity is further augmented by the need to cross the BBB. Here, we presented some platforms that have been engineered to induce a preferential uptake of EV by the BBB vessels, favoring BBB crossing and tumor cell targeting. Engineered exosomes have been produced either to exert a direct cytotoxic effect or to counteract an immunosuppressive microenvironment. This latter can be obtained by using and possibly combining different strategies, since engineered exosomes can deliver chemotherapeutic agents as well as nucleic acids.

Despite the potential, many issues remain to be resolved. Therapeutic use requires very high numbers of exosomes whose pharmacokinetic and pharmacodynamic properties are known and controllable and which are produced according to the guidelines of products intended for clinical use. To date, it has been shown that the exosomes that have the greatest ability to target gliomas are precisely those produced by the tumor cells themselves. Consequently, GDEs will no longer be able to induce proliferation, migration and invasion in the brain parenchyma, but they will be able to load the drug content into tumor cells more efficiently by successfully overcoming the blood–brain barrier.

For this reason, despite the efforts, researchers still did not reach the phase of clinical trials. Moreover, in vitro and in vivo preclinical evidence is needed to find the best and safest method to use GDEs as a therapeutic adjuvant in GBM treatment. A further level of difficulty will reside in the need for a large-scale creation of clinical-grade products.

Nonetheless, the evidence obtained so far deserves to be further expanded to broaden the therapeutic possibilities for glioblastoma, a highly lethal tumor with still very few therapeutic options.

## Figures and Tables

**Table 1:** Summary table of the articles reported below: exosome isolation and loading techniques, type of modification, major effect and level of evidence in vitro and in vivo.

Type of Exosome Modification	Exosome Source	Isolation Techniques	Modification Techniques	Effects of Modified Exosomes	Experimental Model	Reference
PTX incorporation	U87 cells	Exo-spin™ exosome purification kit	Cargo loading by incubation at 37 °C or sonication	PTX loaded exosomes showed an increased cytotoxic effect against GBM cells with respect to free PTX solution	In vitro	[158]
PTX and DOX incorporation	SF776 cells	Ultracentrifugation	Cargo loading by microfluidic device (Exo-Load)	The loading efficiency of DOX and PTX into SF7761 stem cells-like was increased via Exo-Load, reducing tumor proliferation	In vitro	[159]
TMZ and EPZ015666 incorporation	Primary human GBM lines	ExoQuick-TC	Cargo loading by direct or indirect incubation	EVs loaded with the two drugs reduced cell proliferation	In vitro	[160]
Selumetinib incorporation	U87 cells	Ultracentrifugation	Cargo loading by electroporation	U87-Selu EXOs induced cell apoptosis, increasing the level of PARP and decreasing the level of Bcl2	In vitro/ In vivo	[161]
Preparation of Exo@TDPs	U251 and U87 cells	Ultracentrifugation	Sonication for drug incorporation; incubation at 37 °C with Ang-2 and CD1333-targeted peptides	Exo@TDPs, transported through the BBB and penetrated deep tumor parenchyma releasing the cargo	In vitro/ In vivo	[162]
Engineered exosomes- conjugated TGF-β and ACTE (Ds@ACTE)	GL261 cells	Ultracentrifugation	Incubation at 4 °C to mix DBCO-DAK-PEG-Tf with Exo-N_3_	Ds@Exo released DOX into GBM cells and downregulated TGF-β expression, reprogramming immunosuppressive microenvironment	In vitro/ In vivo	[163]
Co-delivery of GDEs with α-galactosylceramide on DC	C6 cells	ExoRNeasy Serum/Plasma Maxi Kit	Incubation at 37 °C for *α*-GalCer incorporation	Co-delivery of GDEs with *α*-GalCer improved TME releasing immunoinhibitory and immunostimulatory factors	In vitro/ In vivo	[164]
Modification of EV surface glycosylation	U87 and primary GBM cells	Ultracentrifugation	Incubation at 37 °C to incorporate palmitoyl-LewisY	Glycan modification reduced immune inhibitory Siglec binding and enhanced EV internalization by DCs in a DC-SIGN dependent manner	In vitro	[165]
Engineered exosomes with ginsenoside (Rg3)	GL261 cells	Ultracentrifugation	Sonication for arsenic trioxide and chlorine e6 incorporation	Platform inhibited tumor growth, induced macrophage polarization and facilitated cytotoxic T lymphocyte infiltration	In vitro/ In vivo	[166]
Engineered exosome-conjugated magnetic nanoparticles (MNPs)	Human mesenchymal stem cells (hMSCs)	Ultracentrifugation	Small interfering RNA (siRNA) of GPX4 (siGPX4) loading by electroporation	Angiopep-2 peptide-modified exosomes penetrated the BBB and targeted GBM cells by recognizing LRP-1 receptors	In vitro/ In vivo	[167]
